# Trophic sympathetic influence weakens pro-contractile role of Cl^−^ channels in rat arteries during postnatal maturation

**DOI:** 10.1038/s41598-020-77092-0

**Published:** 2020-11-17

**Authors:** Daria S. Kostyunina, Lin Zhang, Anastasia A. Shvetsova, Ekaterina K. Selivanova, Olga S. Tarasova, Vladimir V. Matchkov, Dina K. Gaynullina

**Affiliations:** 1 Department of Human and Animal Physiology, Lomonosov Moscow State University, Leninskie Gory 1-12, Moscow, Russia 119234; 2 Department of Biomedicine, Aarhus University, Aarhus, Denmark; 3 Institute for Biomedical Problems, Russian Academy of Sciences, Moscow, Russia; 4 Present Address: School of Medicine, Conway Institute, University College Dublin, Dublin, Ireland; 5 Present Address: Exercise Physiology, Teaching Experiment Center, No.48, Beijing Sport University, Haidian District, Room 403, Xinxi Road, Beijing, 100084 China

**Keywords:** Circulation, Physiology, Cardiovascular biology

## Abstract

Membrane transporters and their functional contribution in vasculature change during early postnatal development. Here we tested the hypothesis that the contribution of Cl^−^ channels to arterial contraction declines during early postnatal development and this decline is associated with the trophic sympathetic influence. Endothelium‐denuded saphenous arteries from 1- to 2-week-old and 2- to 3-month-old male rats were used. Arterial contraction was assessed in the isometric myograph, in some experiments combined with measurements of membrane potential. mRNA and protein levels were determined by qPCR and Western blot. Sympathectomy was performed by treatment with guanethidine from the first postnatal day until 8–9-week age. Cl^−^ substitution in the solution as well as Cl^−^-channel blockers (MONNA, DIDS) had larger suppressive effect on the methoxamine-induced arterial contraction and methoxamine-induced depolarization of smooth muscle cells in 1- to 2-week-old compared to 2- to 3-month-old rats. Vasculature of younger group demonstrated elevated expression levels of TMEM16A and bestrophin 3. Chronic sympathectomy increased Cl^−^ contribution to arterial contraction in 2-month-old rats that was associated with an increased TMEM16A expression level. Our study demonstrates that contribution of Cl^−^ channels to agonist-induced arterial contraction and depolarization decreases during postnatal development. This postnatal decline is associated with sympathetic nerves development.

## Introduction

Chloride (Cl^−^) is an important inorganic anion in the body, which is involved in the regulation of many cellular functions including arterial contraction. In most smooth muscles, Cl^−^ is actively accumulated intracellularly, thereby setting the level of Cl^−^ equilibrium potential above the resting membrane potential^1^. There are several types of Cl^−^ channels in the vascular wall, including voltage-activated Cl^−^ channels (ClC family), volume-regulated anion channels, the cystic fibrosis transmembrane conductance regulator (CFTR) and Ca^2+^-activated Cl^−^-channels (CaCCs)^2,3^. The molecular identity of CaCCs is still uncertain, however, TMEM16A and bestrophin proteins are considered to be essential for Cl^−^ conductance^4,5^.

Agonist-induced smooth muscle cell activation (e.g. by noradrenaline via α_1_-adrenoceptors) causes Ca^2+^ release from intracellular stores, Ca^2+^ influx through channels in cell membrane and sensitization of contractile apparatus to Ca^2+^. All these mechanisms lead to the contraction of smooth muscle cells. Elevation of intracellular Ca^2+^ concentration can activate the CaCCs that are suggested to have a strong contribution to the arterial contraction^6,7^. Opening of the Cl^−^ channels leads to Cl^−^ efflux from smooth muscle cells, which depolarizes the membrane and thus, potentiates voltage-dependent Ca^2+^ influx and contraction.

During early ontogenesis, mammalian circulatory system undergoes significant structural and functional remodeling. It includes the postnatal decrease in the sensitivity of smooth muscle contractile apparatus to Ca^2+^ and alterations in K^+^ channel contribution to the arterial function^8–12^. Until recently, postnatal changes in Cl^−^ homeostasis have not been described for the vascular system, but they were reported for the central nervous system.

In neurons, the intracellular Cl^−^ concentration is increased in early postnatal period compared to an adult organism^13^ due to high expression level of NKCC1 (Na^+^–K^+^–Cl^−^—cotransporter-1, mediates Cl^−^ influx) and low level of KCC2 (a K^+^–Cl^−^—cotransporter, which extrudes Cl^−^ from the cell)^14^. Therefore, activation of GABA_A_ receptors in immature neurons causes Cl^−^ efflux and depolarization, while in mature neurons GABA_A_ receptors mediate Cl^−^ influx and hyperpolarization^13^. We have prevoiusly demonstrated that the contribution of Cl^−^ to arterial contraction decreases during postnatal maturation of vasculature^15^. However, the role of Cl^−^ channels in these changes and the mechanisms behind it remain to be elucidated.

As such a mechanism, we propose here that postnatal reduction in the contribution of Cl^−^ conductance to arterial contraction is associated with trophic influence of sympathetic nerves. This suggestion is based on several findings: (1) the development of sympathetic vasomotor innervation occurs during the first postnatal month in rats^8,16^; (2) sympathetic nerves are known to have trophic influence on arterial structure and function^8,17,18^; (3) sympathetic nerves modulate the expression and activity of several ion channels and pumps^19–22^; (4) TMEM16A protein and sympathetic nerves exhibit opposite expression gradients along the vascular tree^23,24^. However, experimental evidence for the trophic influences of vascular sympathetic innervation on Cl^−^ channel expression and/or function is not available yet.

Thus, in the present study, we tested the hypothesis that the contribution of Cl^−^ channels to arterial contraction declines during early postnatal development and this decline is associated with the trophic influence of sympathetic nerves.

## Results

Normalized inner diameter of relaxed saphenous arteries was 636 ± 99 µm and 274 ± 40 µm in 2- to 3-month-old and 1- to 2-week-old rats, respectively (n = 59, 67; *p* < 0.05). The maximum active wall tension was higher in 2- to 3-month-old rats compared to 1- to 2-week-old rats (8.7 ± 2.2 N/m (n = 59) and 1.8 ± 0.8 N/m (n = 67; *p* < 0.05), respectively) due to larger number of smooth muscle cell layers in their vascular wall^25^.

### Cl^−^ substitution suppresses arterial contraction in 1- to 2-week-old rats stronger than in 2- to 3-month-old rats

**Figure 1:**
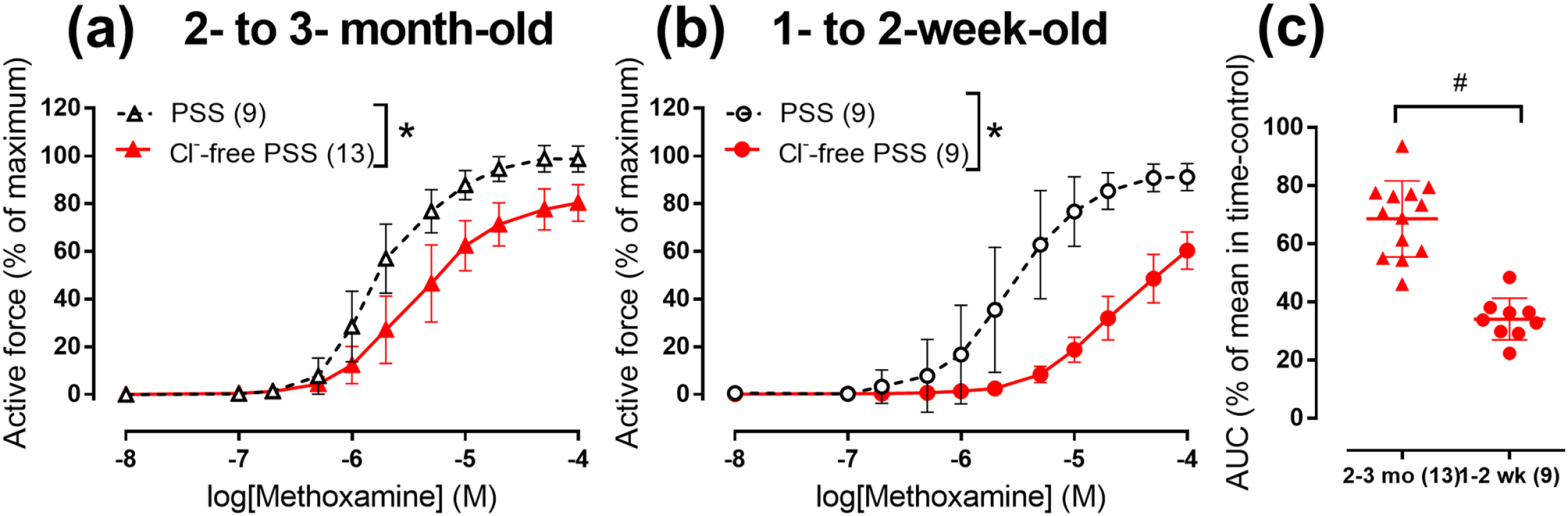
Cl^−^-free PSS suppresses arterial contraction to methoxamine in 1- to 2-week-old rats stronger than in 2- to 3-month-old rats. (**a**,**b**) Concentration–response curves for methoxamine in normal PSS (black symbols) or Cl^−^-free PSS (red symbols) of endothelium-denuded arteries from 2- to 3-month-old (**a**) and 1- to 2-week-old rats (**b**). (**c**) Areas under individual CRCs for methoxamine in Cl^−^-free PSS in (**a**) and (**b**) expressed as percentage of the mean value in the corresponding time-control group. *p < 0.05 (Repeated measures ANOVA), ^#^*p* < 0.05 (Student’s t-test). Numbers in parentheses indicate the number of rats.

In the arteries from 2- to 3-month-old rats as well as from 1- to 2-week-old rats the responses to α_1_-adrenoceptor agonist methoxamine were significantly reduced in Cl^−^ free physiological salt solution (PSS) in comparison with normal PSS (Fig. 1). Substitution of bath Cl^−^ reduced area under concentration–response curve (CRC) to 34 ± 2% (n = 13) in 1- to 2-week-old and to 69 ± 4% (n = 9) in 2- to 3-month-old rats (*p* < 0.05). Thus, reduction of bath Cl^−^ supressed arterial contraction stronger in 1- to 2-week-old rats compared to 2- to 3-month-old rats.

### Cl^−^ substitution depolarizes membrane potential but does not affect intracellular Ca^2+^

In normal PSS, smooth muscle cell membrane potential in endothelium-denuded arteries was less negative in 1- to 2-week-old rats compared to 2- to 3-month-old rats (− 42.6 ± 8.6 mV (n = 17) and − 57.7 ± 6.6 mV (n = 13), respectively, *p* < 0.05). In Cl^−^-free PSS, membrane potential was less negative in comparison with normal PSS in both age-groups (Table 1). Surprisingly, this significant depolarization was followed only by moderate smooth muscle contraction in both 2- to 3-month-old and 1- to 2-week-old rats (Table 1).

**Table 1:** Active force, membrane potential and pH_i_ in endothelium-denuded arteries of 1- to 2-week-old and 2- to 3-month-old rats in normal and Cl^−^-free PSS.

Parameters	2- to 3-month-old rats		1- to 2-week-old rats	
	PSS	Cl^−^-free PSS	PSS	Cl^−^-free PSS
Active force, % of maximum	− 0.3 ± 0.4 (7)	4.2 ± 4.0 (7)^#^	1.1 ± 7.7 (10)	8.1 ± 5.4 (8)^#^
Membrane potential, mV	− 55.7 ± 7.9 (7)	− 34.8 ± 5.5 (7)^#^	− 44.9 ± 11.2 (10)	− 23.4 ± 5.0 (8)^#^
pH_i_	7.44 ± 0.14 (6)	7.64 ± 0.15 (6)^#^	7.39 ± 0.13 (9)	7.61 ± 0.10 (9) ^#^

Data are shown as mean ± SD. Numbers in the parentheses indicate the number of rats. Active force values in each of the experimental conditions were calculated as percentage of the maximum obtained during the CRC for methoxamine.

^#^*p* < 0.05 Cl^−^-free PSS versus PSS in the corresponding group (unpaired Student’s t-test).

Accordingly, intracellular Ca^2+^ in the arteries from 2- to 3-month-old rats was not significantly different under control and in Cl^−^-free conditions (Fura-2 emission ratio values were 0.24 ± 0.03 a.u. and 0.25 ± 0.03 a.u., respectively, n = 6; 6; *p* > 0.05). Therefore, depolarization induced by Cl^−^ substitution was not associated with significant elevation of intracellular Ca^2+^ and contraction.

There was also no difference between intracellular pH (pH_i_) in smooth muscle cells of two age-groups. In normal PSS pH_i_ was similar between 2- to 3-month-old and 1- to 2-week-old rats (Table 1). Substitution of Cl^−^ in PSS significantly alkalized smooth muscles in the arteries from both 2- to 3-month-old and 1- to 2-week-old rats (Table 1). However, pH_i_ in Cl^−^-free solution was the same in both age-groups (Table 1).

### Cl^−^ substitution abolishes methoxamine-induced depolarization in 1- to 2-week-old but not in 2- to 3-month-old rats

**Figure 2:**
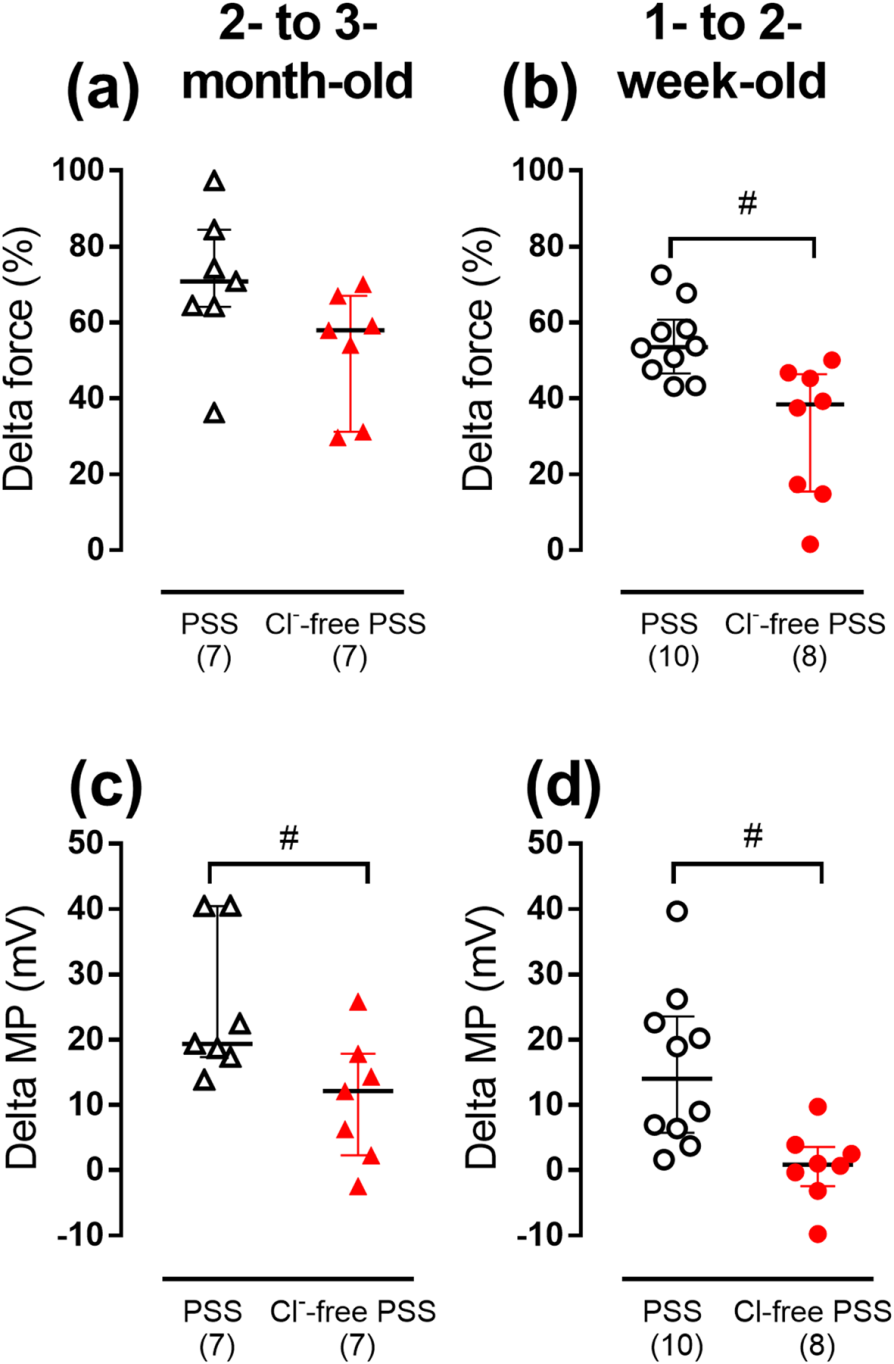
The effects of Cl^−^-substitution on methoxamine-induced changes of force and membrane potential (MP) of smooth muscle cells in endothelium-denuded arteries from two age-groups of rats. Methoxamine-induced changes of force (**a**,**b**) and membrane potential (**c**,**d**) in normal or Cl^−^-free PSS in arteries from 2- to 3-month-old (**a**,**c**) and 1- to 2-week-old rats (**b**,**d**). Data are shown as median and interquartile range. ^#^ *p* < 0.05 (Mann Whitney U test). Numbers in parentheses indicate the number of rats.

In normal PSS methoxamine-induced arterial contraction was associated with smooth muscle cell depolarization in both age-groups (Fig. 2). However, in Cl^−^-free PSS, methoxamine-induced contraction was associated with smooth muscle cell depolarization only in the arteries from 2- to 3-month-old rats (Fig. 2a,c), but not in 1- to 2-week-old rats (Fig. 2b,d). In 2- to 3-month-old group methoxamine (10 µM) changed membrane potential from − 34.8 ± 5.5 mV to − 23.9 ± 5.1 mV (n = 7, *p* < 0.05). In 1- to 2-week-old group, the values of membrane potential at the baseline and in the presence of 10 µM methoxamine were − 23.4 ± 5.0 mV and − 22.8 ± 5.1 mV, respectively (n = 8, p > 0.05). Thus, our data indicate that both methoxamine-induced depolarization and contraction are more Cl^−^-dependent in the arteries from 1- to 2-week-old rats compared to 2- to 3-month-old animals.

### The effect of Cl^−^ channel blockers on arterial contraction is stronger in 1- to 2-week-old rats than in 2- to 3-month-old rats

**Figure 3:**
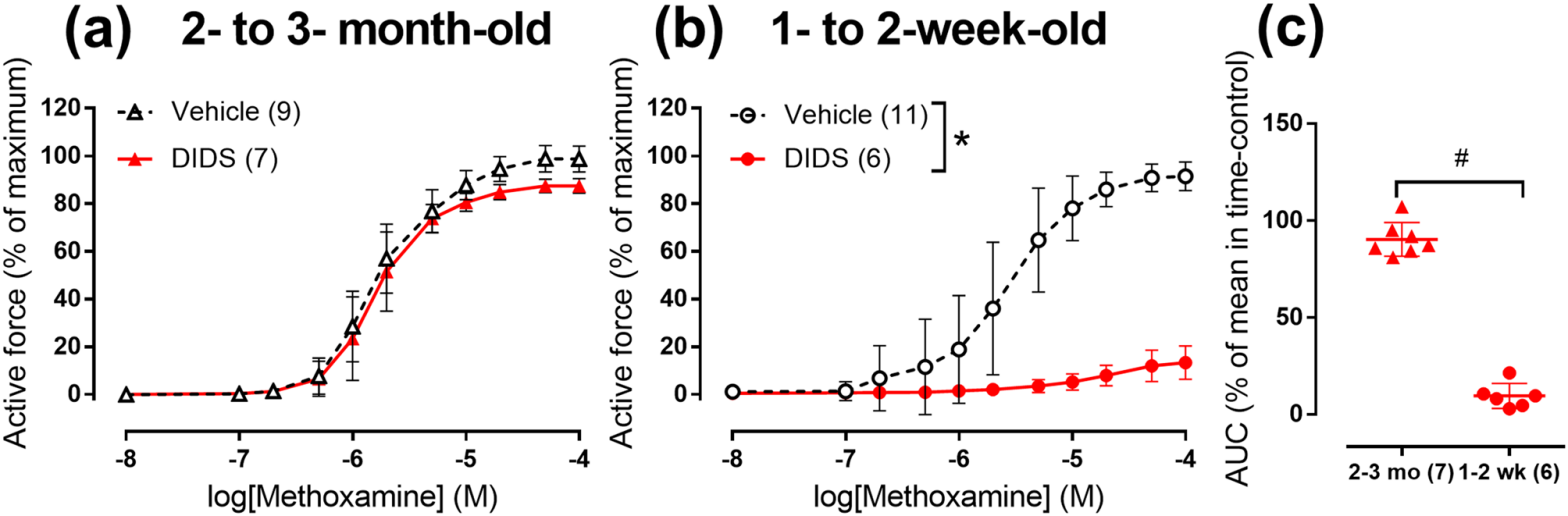
Effects of 1 mM DIDS on contractile responses to methoxamine of endothelium-denuded arteries from two age-groups of rats. (**a**,**b**) CRCs to methoxamine in the presence of vehicle (black symbols) or 1 mM DIDS (red symbols) of arteries from 2- to 3-month-old (**a**) and 1- to 2-week-old rats (**b**). (**c**) Areas under individual CRCs to methoxamine in the presence of 1 mM DIDS in (**a**) and (**b**) expressed as percentage of mean value in the corresponding group exposed to vehicle. *p < 0.05 (Repeated measures ANOVA), ^#^ *p* < 0.05 (Student’s t-test). Numbers in parentheses indicate the number of rats.

In order to identify whether the elevated Cl^−^ contribution to vasocontraction in 1- to 2-week-old rats is associated with greater impact of Cl^−^ channels, we inhibited Cl^−^ membrane transport with 1 mM DIDS^26^. DIDS did not affect arterial contraction to methoxamine in 2- to 3-month-old rats (Fig. 3a) but almost completely abolished contraction in 1- to 2-week-old rats (Fig. 3b,c). This suggests larger contribution of Cl^−^ transport to arterial responses of 1- to 2-week-old rats.

**Figure 4:**
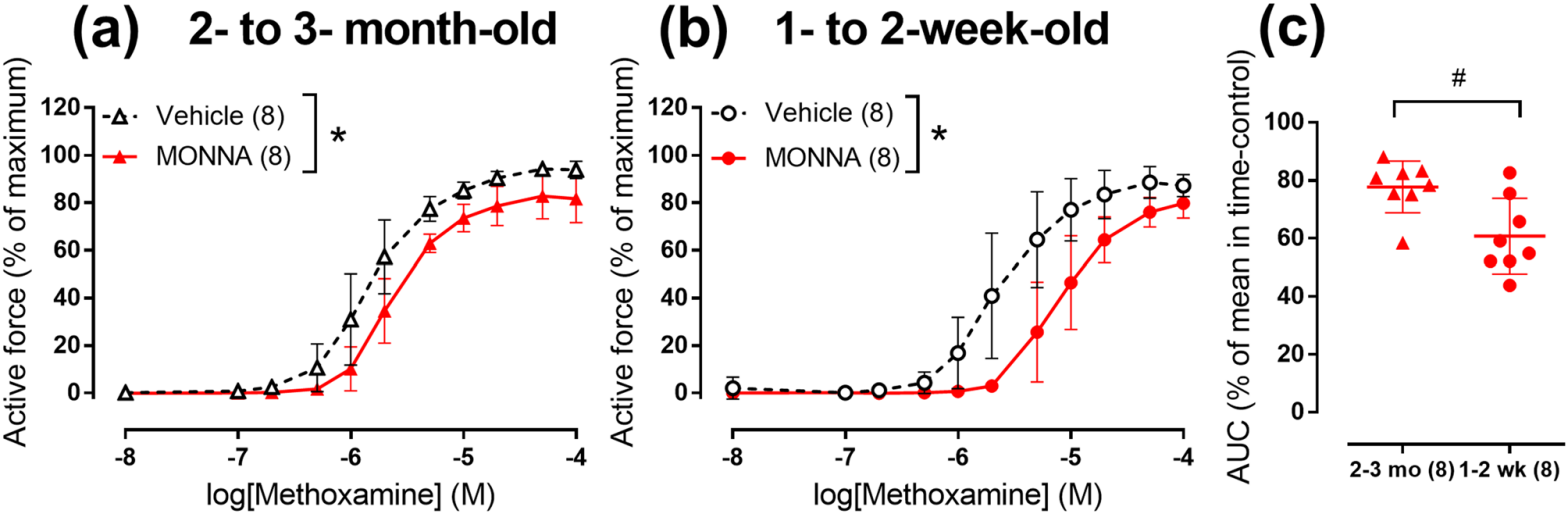
TMEM16A blocker MONNA suppresses arterial contraction stronger in 1- to 2-week-old rats in comparison with 2- to 3-month-old rats. (a-b) CRCs to methoxamine in the presence of vehicle (black symbols) or 3 μM MONNA (red symbols) of endothelium-denuded arteries from 2- to 3-month-old (**a**) and 1- to 2-week-old (**b**) rats. (**c**) Areas under individual CRCs to methoxamine in the presence of 3 μM MONNA in (**a**) and (**b**) expressed as percentage of mean value in the corresponding group exposed to vehicle. *p < 0.05 (Repeated measures ANOVA), # *p* < 0.05 (Student’s t-test). Numbers in parentheses indicate the number of rats.

MONNA was previously reported as a putative TMEM16A channel blocker^27^ . In our study 3 μM MONNA reduced arterial contraction in both 2- to 3-month-old (Fig. 4a, Supplementary Figure S1a) and 1- to 2-week-old (Fig. 4b, Supplementary Figure S1b) rats. MONNA reduced area under CRC to 61 ± 5% (n = 8) in 1- to 2-week-old and only to 78 ± 3% (n = 8) in 2- to 3-month-old rats (*p* < 0.05, Fig. 4c). Thus, the effect of MONNA was also more pronounced in arteries of 1- to 2-week-old rats compared to 2- to 3-month-old rats.

### The abundance of Cl^−^ channels is higher in the arteries from 1- to 2-week-old rats than in 2- to 3-month-old rats

**Figure 5:**
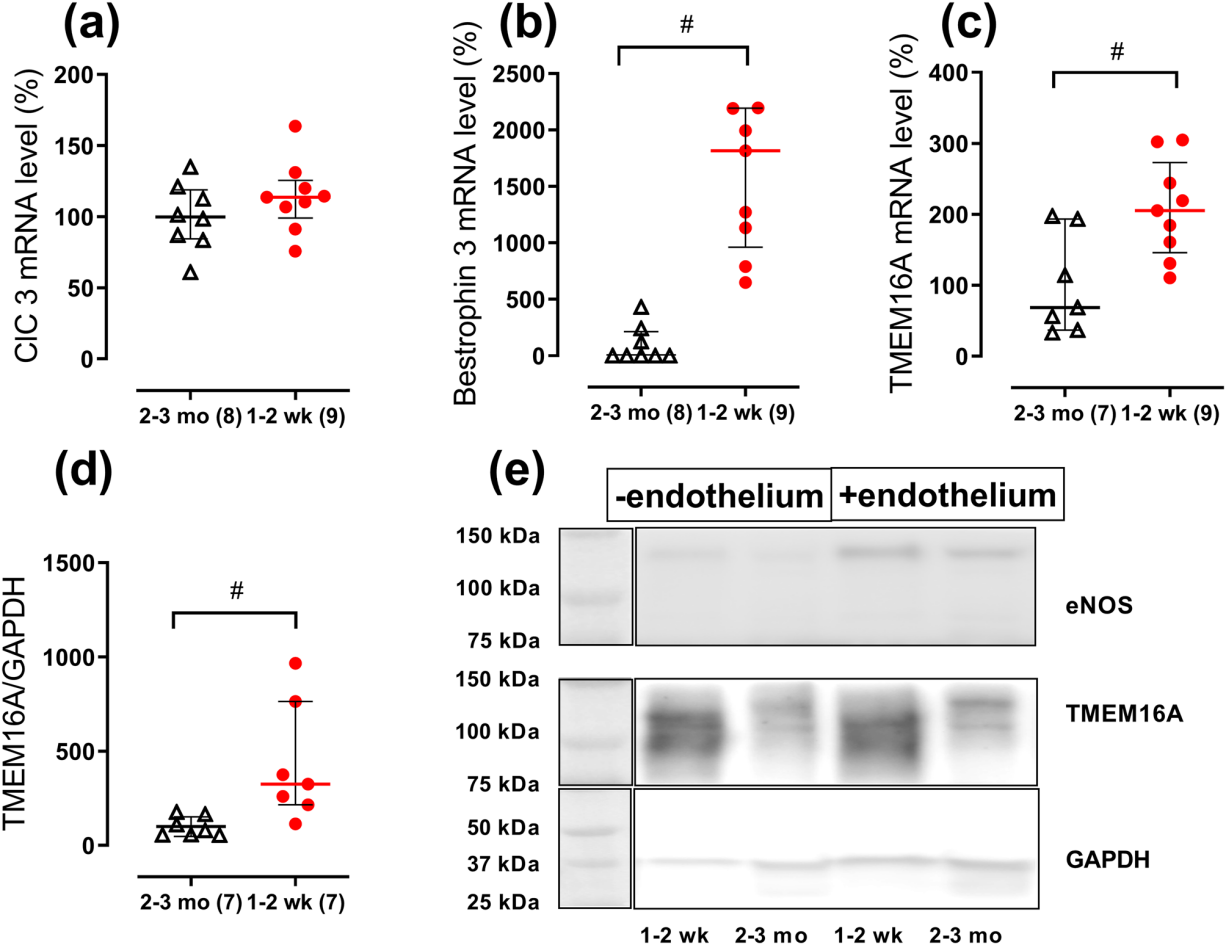
Expression of Cl^−^ channels in endothelium-denuded arteries in two age-groups of rats. (**a**–**c**) Relative mRNA levels of ClC 3 (**a**), bestrophin 3 (**b**) and TMEM16A (**c**) in arteries of 2- to 3-month-old and 1- to 2-week-old rats. (**d**) Relative protein level of TMEM16A in arteries from 2- to 3-month-old and 1- to 2-week-old rats. (**e**) Representative Western blot for two arteries from 1- to 2-week-old rats and two arteries from 2- to 3-month-old. The blot was divided into upper parts stained with eNOS antibody as a control for endothelium removal or with TMEM16A antibody, and lower part stained with GAPDH antibody to control for protein loading, as indicated. Full-length blots are presented in Supplementary Figure S2. All data are expressed as the percentage of mean value of the 2- to 3-month-old group and shown as median and interquartile range. ^#^ *p* < 0.05 (Mann Whitney U test). Numbers in parentheses indicate the number of tissue samples.

To explore the molecular background for larger contribution of Cl^−^ transport to arterial contraction in 1- to 2-week-old rats, we studied the expression of Cl^−^ channels in endothelium-denuded arteries from 2- to 3-month-old and 1- to 2-week-old rats. The mRNA expression level of ClC3 was not different in the arteries from two age-groups (Fig. 5a). CFTR mRNA was not detected in saphenous artery of either 2- to 3-month-old or 1- to 2-week-old rats though primer’s efficiency was confirmed using rat testis.

In accordance to our functional findings, mRNA content of the CaCC-associated proteins, TMEM16A and bestrophin 3, was significantly higher in endothelium-denuded arteries of 1- to 2-week-old rats compared to 2- to 3-month-old rats (Fig. 5b,c). This was further supported by Western blot showing an increased protein abundance of TMEM16A in endothelium-denuded arteries from 1- to 2-week-old rats compared to 2- to 3-month-old rats (Fig. 5d,e).

### Chronic sympathetic denervation preserved larger Cl^−^ contribution to the arterial contraction in 2- to 3-month-old rats

To test our hypothesis that postnatal decline in Cl^−^ contribution is associated with trophic influences of sympathetic nerves, we compared the contribution of Cl^−^ to arterial contraction in 2-month-old chronically sympathectomized and control rats. Rats treated with guanethidine were characterized by decreased body weight (179 ± 35 g (n = 9) and 263 ± 26 g (n = 8) for sympathectomized and control groups, respectively, *p* < 0.05). Adrenergic nerve plexus was not observed in saphenous artery of sympathectomized rats in contrast to control group suggesting successful sympathetic denervation (Supplementary Figure S3). Saphenous arteries of sympathectomized rats had smaller inner relaxed diameter than control group (454 ± 39 µm (n = 8) and 595 ± 37 µm (n = 8), respectively, *p* < 0.05) as well as maximum active wall tension (6.1 ± 0.8 N/m (n = 8) and 9.1 ± 1.1 N/m (n = 8), *p* < 0.05). The sensitivity to methoxamine was estimated by calculating pD_2_ values that were higher in sympathectomized group compared to the control group (5.83 ± 0.18 (n = 8) and 5.54 ± 0.24 (n = 8), respectively, *p* < 0.05).

**Figure 6:**
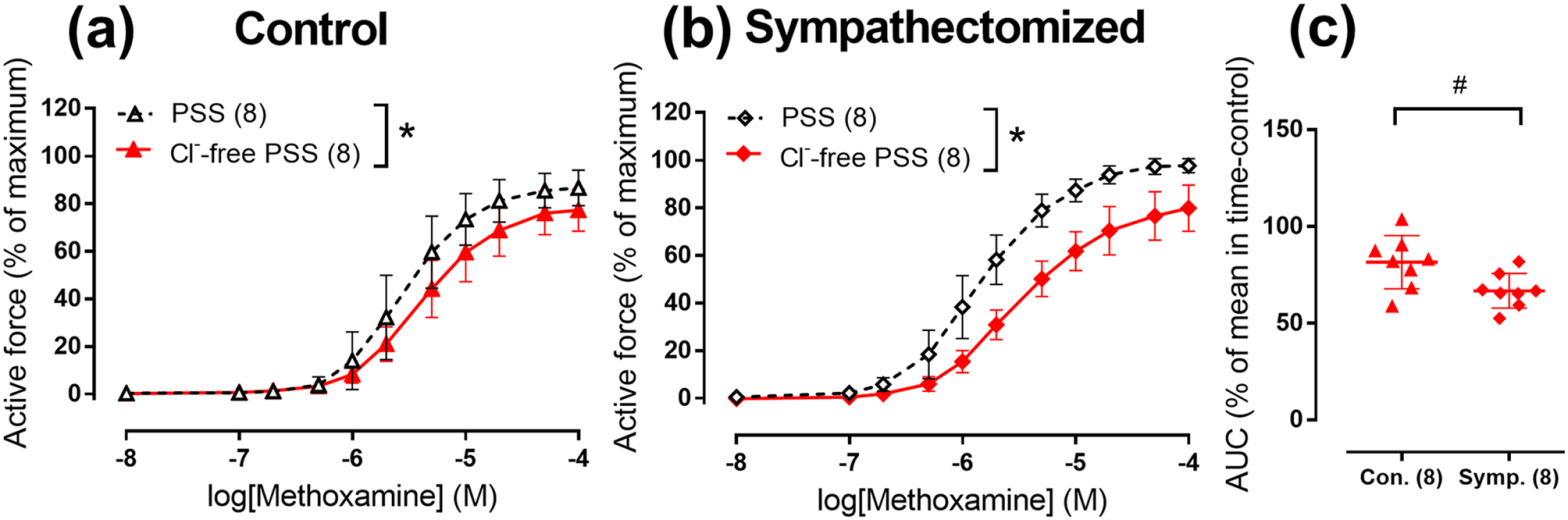
Cl^−^-free PSS suppresses arterial contraction to methoxamine in neonatally sympathectomized rats stronger than in rats with intact sympathetic nervous system. (**a**,**b**) CRC for methoxamine in normal PSS (black symbols) or Cl^−^-free PSS (red symbols) of endothelium-denuded arteries from control (**a**) and sympathectomized rats (**b**). (**c**) Areas under individual CRCs for methoxamine in Cl^−^-free PSS in (**a**) and (**b**) expressed as percentage of the mean value in the corresponding time-control group. *p < 0.05 (Repeated measures ANOVA), # *p* < 0.05 (Student’s t-test). Numbers in parentheses indicate the number of rats.

In Cl^−^-free PSS, the arterial contractile responses were reduced in both control (Fig. 6a) and sympathectomized (Fig. 6b) groups compared to normal PSS. Importantly, Cl^−^ substitution reduced area under CRC in sympathectomized group more than in control group (Fig. 6c) suggesting that Cl^−^ contribution to the contraction is increased in chronically denervated arteries.

**Figure 7:**
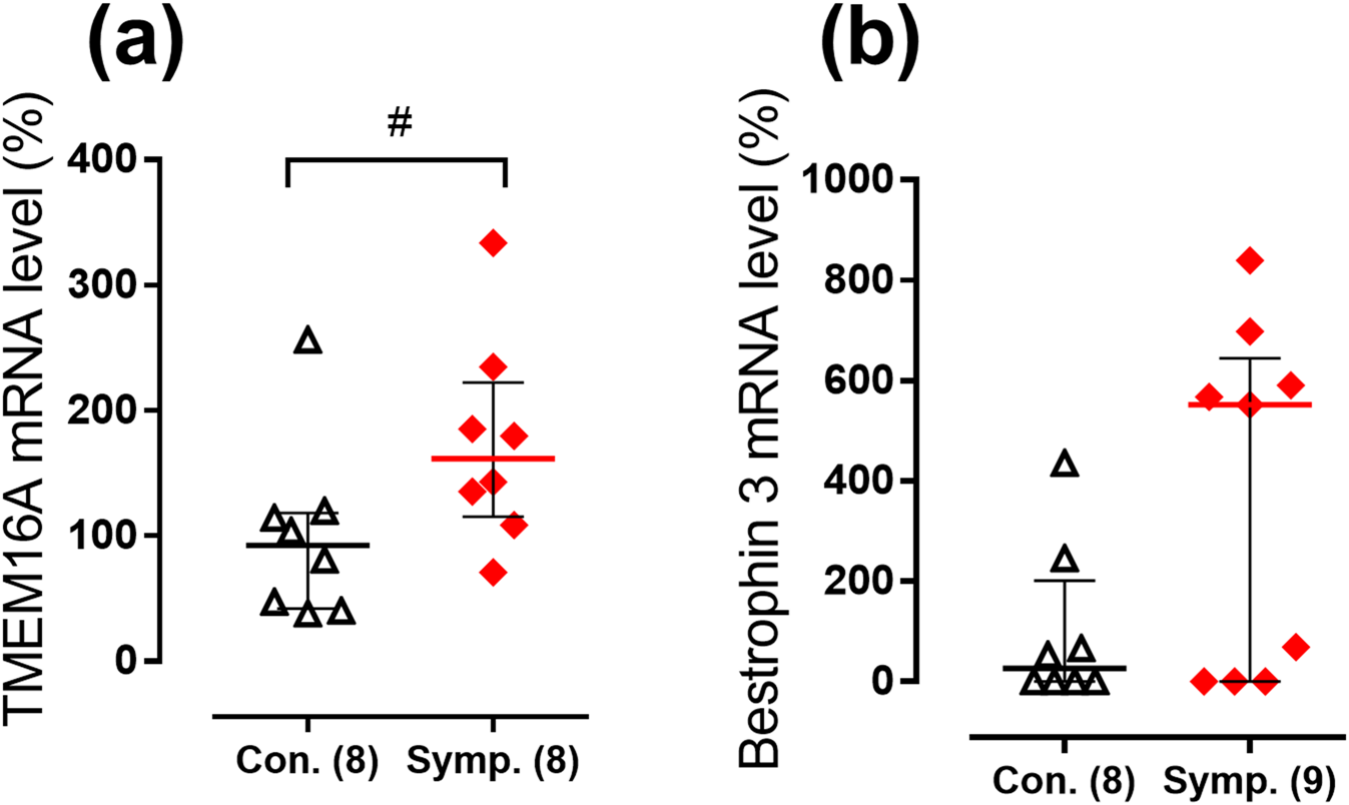
Relative expression levels of TMEM16A (**a**) and bestrophin 3 (**b**) mRNA in endothelium-denuded arteries from neonatally sympathectomized rats (Symp.) in comparison to rats with intact sympathetic nervous system (Con.). Data are expressed as the percentage of mean value of the control group and shown as median and interquartile range. #*p* < 0.05 (Mann Whitney U test). Numbers in parentheses indicate the number of tissue samples. For samples where the rise of fluorescence signal was not detected before cycle 40 of qPCR reaction mRNA expression level was considered as zero.

Furthermore, to get an insight into the molecular background for augmented contribution of Cl^−^ to the regulation of arterial contraction in sympathectomized group, we looked at mRNA expression levels of TMEM16A and bestrophin 3. The expression of TMEM16A was larger in sympathectomized group compared to control group (Fig. 7a), while the difference in bestrophin 3 mRNA levels did not achieve significance (Fig. 7b).

## Discussion

Here we studied the developmental alterations in the depolarizing and pro-contractile role of Cl^−^ in peripheral artery of systemic circulation. Arteries of 1- to 2-week-old rats demonstrated higher contribution of Cl^−^ conductance to the regulation of arterial contraction as compared to 2- to 3-month-old rats. This was also suggested by larger effects of Cl^−^ substitution, DIDS and MONNA in the arteries of younger age-group. Moreover, we showed that vascular smooth muscle cells of 1- to 2-week-old rats contained larger quantity of TMEM16A and bestrophin 3 in comparison to 2- to 3-month-old group. Finally, we revealed that chronic sympathetic denervation prevents postnatal decline of Cl^−^ contribution to the contraction in rat arteries.

Cl^−^ substitution in PSS reduced arterial contraction to methoxamine in the arteries from 2- to 3-month-old rats. This is in accordance with previous findings showing reduced sensitivity to noradrenaline of mesenteric arteries from adult rats and mice in Cl^−^-free PSS^28^. Moreover, in the present study, we evaluated the arterial contraction of 1- to 2-week-old rats in Cl^−^-free PSS and found that it was markedly suppressed compared to normal PSS. This result is in accordance with our previous observation in another study^15^. Importantly, the reduction of contraction in Cl^−^-free PSS was larger in the arteries of 1- to 2-week-old rats compared to adult rats.

Notably, substitution of Cl^−^ ions in PSS by aspartate caused considerable depolarization of arterial smooth muscle cells from both 1- to 2-week-old and adult rats. Accordantly, previous data reported that Cl^−^ substitution by aspartate or sulfate salts depolarized rat mesenteric arteries^28^. Intriguingly, substitution of Cl^−^ depolarized the arteries from both age-groups but did not cause severe increase in vascular tone (an elevation of tone was appr. 4% and 8% of maximum active force for 2- to 3-month-old and 1- to 2-week-old rats, respectively). Accordantly, the depolarization was not accompanied by an increase of intracellular Ca^2+^ in vascular smooth muscle cells. This can be due to change of pH_i_ that accompanies substitution of extracellular Cl^−^ in arteries of both 1- to 2-week-old and 2- to 3-month-old rats. Our results on pH_i_ alkalization due to substitution of extracellular Cl^−^ are in accordance with previous reports^29^. These changes of pH_i_ might in turn modulate enzymatic activity in smooth muscle cells^30^. However, the exact mechanism responsible for inhibition of contraction despite strong depolarization of smooth muscle cells is unknown and beyond the scope of the present study. Importantly, an alkalization in response to Cl^−^ substitution was observed in both age-groups and was similar between them. Thus, this cannot explain the difference in methoxamine-induced contractions.

The importance of extracellular Cl^−^ ions for depolarization of arterial smooth muscle cells is also significantly larger in 1- to 2-week-old than in 2- to 3-month-old rats. In arteries of 2- to 3-month-old rats, methoxamine-induced depolarization was decreased in Cl^−^-free PSS. Similarly, in rat mesenteric arteries noradrenaline-induced depolarization was reduced in Cl^−^-free PSS^28^. In contrast to arteries of adult rats, in the arteries of younger rats substitution of Cl^−^ completely abolished depolarization. We did not measure the Cl^−^ currents in smooth muscle cells of 1- to 2-week-old rats in order to compare the Cl^−^ conductance between the different age-groups. However, our membrane potential measurements suggest that transmembrane Cl^−^ ion gradient is essential for agonist-induced depolarization and contraction of arteries from 1- to 2-week-old rats. A higher mRNA expression of NKCC1 might point to the increased Cl^−^ influx in smooth muscle cells of 1- to 2-week-old compared to 2- to 3-month-old rats (Supplementary figure S4) but functional significance of this difference needs to be validated in future studies.

We further tested the contribution of Cl^−^ conductance to the arterial contraction pharmacologically. We used two different blockers, DIDS and MONNA. DIDS is a conventional blocker widely used to inhibit Cl^−^ conductance^31,32^. Structurally different from DIDS, MONNA was suggested to be a putative TMEM16A channel blocker^27^. Importantly, both blockers showed stronger effects in the arteries of 1- to 2-week-old rats compared to 2- to 3-month-old animals*.*

The pharmacology of Cl^−^ membrane transport is relatively poor. DIDS was shown to inhibit other membrane transporters including anion exchanger AE2^33^, K_ATP_ channels^34^ and Ca^2+^-ATPase^35^. Similarly, MONNA was reported to have Cl^−^-independent vasorelaxing effects in the arterial wall^28^. However, in this study, the effects of both blockers were qualitatively similar to the effects of Cl^−^-free PSS. Based on these independent approaches, we concluded that the contribution of Cl^−^ channels to arterial contraction is higher in the arteries of 1- to 2-week-old rats compared to 2- to 3-month-old rats.

The molecular background of augmented contribution of Cl^−^ conductance in 1- to 2-week-old rats was addressed by comparing the expression of Cl^−^ channels in these two age-groups. In accordance with our functional data, smooth muscle cell expression of TMEM16A and bestrophin 3 was considerably higher in 1- to 2-week-old rats than in 2- to 3-month-old rats.

Two antibody-stained bands seen in TMEM16A Western blot may be a result of either different splice variants or post-transcriptional modification of the protein. In fact, TMEM16A was shown to have many splice variants in different tissues^36^, including vasculature^37^. TMEM16A is known to be essentially important for Ca^2+^-activated Cl^−^ conductance in vascular smooth muscle cells^37–40^ although its specific contribution to Cl^−^ channel formation remains uncertain^4^. In accordance with previous observations the expression of TMEM16A and bestrophin 3 is interdependent i.e. the expression of one protein can affect another protein’s expression^38^. We suggest similar relation in the early postnatal development where an augmented expression of TMEM16A in the arteries from 1- to 2-week-old rats leads to an upregulation of bestrophin 3. However, the mechanism and importance of this interdependence remains to be elucidated.

To the best of our knowledge, the relative expression of CaCCs has never been explored in developing arteries. However, higher TMEM16A expression during early stages of development was previously described in olfactory epithelium of mice^41^. Moreover, larger abundance of bestrophin family member, bestrophin 1, was shown in murine eyes in early postnatal development^42^. Thus, high level of CaCCs in early postnatal period discovered in the present study is not the unique feature of immature smooth muscle cells and may accompany the development of different tissues.

Sympathetic nerves are well-known to have trophic influence on arterial structure and function^17,18^. Arterial sympathetic innervation develops during the first month of postnatal development in rats^8,16^. In order to identify the trophic influence of sympathetic nerves, we prevented their development by treating rats with guanethidine from the first postnatal day until the age of 8–9 weeks. Despite delayed growth rate, sympathectomized rats did not demonstrate any alterations in behavior activity or health status, which is in line with previously reported data^43^. Chronic sympathetic denervation increased smooth muscle sensitivity of agonist-induced contraction. This is in accordance with previous findings showing the increase in arterial sensitivity to contractile stimuli^21,44^.

Our functional data with Cl^−^-free PSS for the first time demonstrate that sympathectomized rats had larger Cl^−^ contribution to the agonist-induced contraction than matched control group. We did not perform experiments with MONNA or DIDS on arteries from sympathectomized rats. However, our suggestion is supported by the fact that larger Cl^−^ contribution to the agonist-induced contraction is associated with the increased level of TMEM16A mRNA in smooth muscle cells of sympathectomized rats compared to control animals. Our results show that development of sympathetic nerves and a change in the Cl^−^ dependence of contraction are related processes. Therefore, we conclude that in accordance with our hypothesis the postnatal declines in Cl^−^ contribution to the regulation of arterial contraction and CaCCs expression are associated with trophic influences of sympathetic nerves but the exact mechanisms are still unknown.

**Figure 8:**
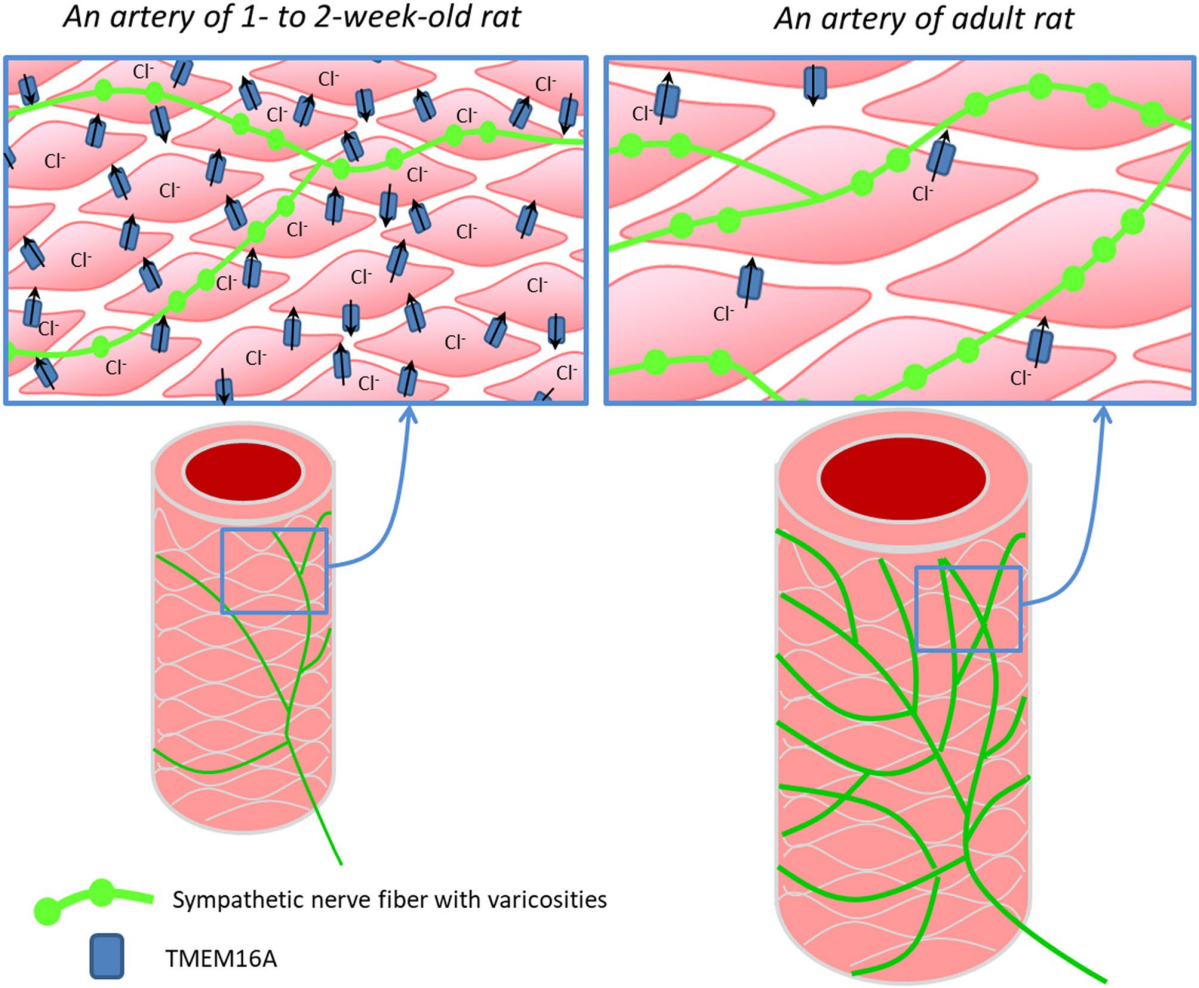
Sympathetic nerves govern postnatal decline in the contribution of Cl^−^ channels to arterial contraction. In the early postnatal period poorly innervated arterial smooth muscle cells contain a larger number of Cl^−^ channels resulting in a large contribution of Cl^−^ to arterial contraction. After maturation, the expression level of Cl^−^ channels in densely innervated vascular wall is relatively low resulting in smaller contribution of Cl^−^ to arterial contraction.

To conclude, our comprehensive study for the first time demonstrates that contribution of Cl^−^ channels to the arterial contraction and agonist-induced depolarization of smooth muscle cells is larger at early postnatal development. This is enabled by higher expression of TMEM16A and bestrophin 3, the molecules responsible for the Ca^2+^-activated Cl^−^ conductance, in 1- to 2-week-old than in 2- to 3-month-old arteries. We suggest that higher contribution of the Ca^2+^-activated Cl^−^ conductance in vasculature of immature organism facilitates their vasoconstriction by counteracting the high activity of many types of K^+^ channels in 1- to 2-week-old rats^12^. Our study uncovered a novel target of trophic influences of sympathetic nerves. We showed for the first time that trophic influences of sympathetic nerves govern postnatal decline in the functional contribution of Cl^−^ channels (Fig. 8).

## Methods

### Animals

Experiments were conducted in accordance with European Convention on the protection of animals used for scientific purposes (EU Directive 2010/63/EU). Approval for the use of laboratory animals and all procedures used in this study was granted by Russian institutional committee on animal welfare (107-g). The experiments were approved by and conducted with permission from the Animal Experiments Inspectorate of the Danish Ministry of Environment and Food no. 2016-15-0201-00982. Rats were sacrificed under CO_2_ narcosis by decapitation.

To study alteration in the contribution of Cl^−^ channels to arterial contraction with age, we used 10- to 14-day-old (referred as 1- to 2-week-old rats) and 2.5- to 3.5-month-old (i.e. 2- to 3-month-old rats) male Wistar rats.

To study the trophic influence of sympathetic nerves, we performed chronic pharmacological sympathectomy^45^. The animals were chronically treated with guanethidine monosulfate (Santa Cruz, dissolved in 0.9% NaCl) starting from the first postnatal day until the age of 8–9 weeks. Rat litters were randomly divided into two groups immediately after birth; sympathectomized group that received injections of guanethidine (n = 6 litters) and control rats (n = 5 litters). Guanethidine was injected subcutaneously 6 days per week in the dose of 25 mg/kg for 2 weeks (1–15 postnatal days) followed by 50 mg/kg (from the 16th postnatal day to 8–9-week age). Control animals received similar volume of 0.9% NaCl (i.e. 1.7–2.5 ml/kg). The injections of guanethidine were performed by skilled researcher using syringes with 30G needles. All rat pups survived and did not show any sign of discomfort during injections. Two days after the last injection, the rats were sacrificed and saphenous arteries were isolated for further wire myograph and qPCR studies. Adrenergic nerve plexus was visualized using glyoxylic acid staining^46^ using Axiovert 200 microscope and S25 filter set.

### Wire myograph experiments

Several 2-mm-long segments of saphenous artery were dissected and mounted in the isometric myograph (Danish Myo Technology (DMT) A/S, Denmark). The endothelium was removed by rubbing inner lumen of the artery with a rat whisker. Force transducer signals were digitalized at 10 Hz and recorded on a PC hard drive using an analogue-to-digital converter (E14-140 M, L-CARD, Russia) and the PowerGraph 3.3 software (DISoft, Russia, https://www.powergraph.ru/). PSS in myograph chamber was warmed to 37° and arterial segments were gradually stretched to 0.9d_100_; d_100_ is an inner diameter of relaxed artery subjected to the transmural pressure of 100 mmHg^47^. A standard start-up procedure consisted of (1) noradrenaline (10 µM) application; (2) an application of acetylcholine (10 µM) to methoxamine (1–3 µM) pre-constricted artery (a test for successful endothelium removal) and (3) an application of methoxamine (10 µM). This start-up procedure enables stable and reproducible vascular responses throughout the experiment.

The experimental protocol included (unless other specified) two CRC for the α_1_-adrenoceptor agonist methoxamine (in the range from 0.01 to 100 µM). The first CRC was performed in normal PSS for all studied segments. Subsequently, some arterial segments were subjected to experimental challenges while other served as a time-control. In part of experiments, the arterial preparations were further incubated for 30 min in Cl^−^-free PSS (for composition see below) or in normal PSS (i.e. time-control). Five to 10 min after beginning of the incubation period the preparations were stimulated with 10 µM of methoxamine for 5 min followed by washout. This was done in order to stimulate Cl^−^ efflux from smooth muscle cells^28^. For pharmacological challenges in another part of experiments, one arterial segment was exposed for 30 min to Cl^−^ channel blocker, either 1 mM DIDS^26,48^ or 3 µM MONNA, while another arterial segment was incubated with a vehicle. The concentration of MONNA 3 µM was selected based on our previous studies, where it caused submaximal relaxation^28^.

Active force values were calculated by subtraction the passive force values recorded in the preparation with fully relaxed smooth muscle (in PSS for vessel isolation, for composition see section “Solutions”, supplemented with NO-donor DEA/NO, 1 µM) from baseline and each methoxamine concentration. All active force values in the second CRC were expressed as the percentage of the maximum active force achieved during the first CRC. In order to compare the effects of experimental challenges in two age-groups, the area under individual second CRCs to methoxamine were calculated in GraphPad Prism 7.0 (La Jolla, CA, USA, https://www.graphpad.com/scientific-software/prism/) and then, expressed as a percentage of the mean value for matched time-control group. The arterial sensitivity to agonist was assessed as pD2 (i.e. the negative logarithm base 10 of EC_50_—the concentration of agonist, which causes a half-maximum effect) that was calculated by fitting the second individual CRCs to sigmoidal function (variable slope) using GraphPad Prism 7.0. pD2 values were obtained for each individual curve and then, their mean and SD values and were calculated for each group.

### Membrane potential measurements

The membrane potential of smooth muscle cells in endothelium-denuded arteries was measured as described previously^28^ using sharp glass microelectrodes (AF100-64-10, World Precision Instruments Ltd, Hitchin, UK) pulled on a horizontal puller (P-97, Sutter Instrument Co., Novato, CA, USA). Microelectrodes were filled with 3 M KCl; their resistance ranged from 30 to 150 MOhm. The resistance of the electrode was continuously monitored by applying subthreshold electrical pulses with 1.1 nA amplitude and 30 ms duration at a frequency of 1 Hz. The reference electrode (flat-tip Ag/AgCl probe; Warner Instruments, Hamden, CT, USA) was placed in the bath solution. The amplifier was zeroed after the recording electrode was immersed in the bath solution. Voltage was amplified (Intra 767, World Precision Instruments Ltd) and recorded simultaneously with isometric force at 1 kHz using PowerLab 4SP system and LabChart 8 software (ADInstruments, Dunedin, New Zealand, https://www.adinstruments.com/).

Membrane potential recordings were accepted according to the following criteria: (1) a sharp drop of the potential on cell penetration; (2) a stable level of the membrane potential for at least 30 s; (3) a return to the zero-potential level after electrode removal; (4) similar electrode resistance values before and after the impalement. The liquid junction potential estimated for the Cl^−^-free PSS was minor (− 1.3 mV; Junction Potential Calculator, Clampex10, Molecular Devices Ltd, Wokingham, UK). This was further supported by control experiments where 3 M KCl salt-agar bridge was placed between the Ag/AgCl pellet and the bath solution without any significant implications for the measurements. We therefore did not correct the data for the junction potential.

A segment of saphenous artery was mounted in a wire myograph (DMT A/S, Denmark). To exclude distortion of the electrode by gas bubbles, warm (37 °C) and equilibrated with 5% CO_2_ in O_2_ PSS was supplied from a separate reservoir to the myograph chamber (10 ml volume) by a peristaltic pump at the rate of 4.5 ml/min. The standard start-up procedure and methoxamine CRC were performed as described above. Then, the solution was changed to Cl^−^-free PSS for at least 30 min (with Cl^−^-depleting activation with 10 µM methoxamine—see above) and membrane potential was measured first in the absence and then in the presence of methoxamine (10 µM). In time-control group similar measurements were done in normal PSS.

### Measurement of mRNA expression levels in arterial tissue by qPCR

Measurement of mRNA expression level was performed as described previously^12^. Saphenous arteries were isolated and cut in approximately 8-mm long segments, which were quickly mounted in an ice-cooled analogue of a wire myograph chamber and endothelium was removed using a rat whisker. Arteries were stored in RNA-later solution (Qiagen) at − 20 °C. RNA was extracted using the kit from Evrogen according to the manufacturer’s instructions with RLT- buffer (Qiagen). All RNA samples were processed with DNase I (Fermentas, 1000 U/mL). RNA concentration was measured by a NanoDrop 1000 (Thermo Scientific, USA) and then all sample concentrations were adjusted to 70 ng/µL by dilution. Reverse transcription was performed using the MMLV RT kit (Evrogen, Russia) according to the manufacturer’s manual. qPCR was run in the RotorGene6000 (Corbett Research, Australia) using qPCRmix-HS SYBR (Evrogen).

All primers used in the study were synthesized by Evrogen, their sequences are listed in Table 2. Gene expression levels were calculated using the RotorGene6000 software. Primer efficiency was identified using the LinRegPCR 2018.0 Software^49^ (www.medischebiologie.nl). The primer efficiencies for all studied genes were in the range 1.8–2.0. The expression level of mRNA was calculated as *E*^*−Ct*^, where *E* is primer efficiency and *Ct* is cycle threshold. These values were normalized to the geometric mean of the two housekeeping RNAs (*Gapdh* and *Rn18s*), detected in the same sample. Data are expressed as the percentage of mean value of the 2- to 3-month-old group and shown as median and interquartile range. Endothelium removal was confirmed by more than 15-fold reduction in eNOS mRNA content in endothelium-denuded versus endothelium-intact preparations (data not shown).

**Table 2:** Primers’ sequences.

Protein name	Gene name	Forward	Reverse
GAPDH	*Gapdh*	CACCAGCATCACCCCATTT	CCATCAAGGACCCCTTCATT
18 s rRNA	*Rn18s*	CACGGGTGACGGGGAATCAG	CGGGTCGGGAGTGGGTAATTTG
ClC3	*Clcn3*	GATCTCAAGCAGATGCGGGG	GAGCTCCTCATCGCTACTCG
TMEM16A	*Ano1*	TGTGATCTCCTTCACGTCTG	CATACTTGTGTTCTGACCACG
Bestrophin 3	*Best3*	CTCATCTCCAGCAGTGTCCA	CAGATGAGGCGACTTGAGG
CFTR	*Cftr*	GCGATGCTTTGTCTGGAGATTC	CCACTTGTAAAGGAGCAATCCATA

### Western blotting

Western blotting experiments were performed as described previously^23^. Endothelium-denuded arterial segments were snap frozen in liquid nitrogen and lysed in ice cold Pierce lysis buffer using a pellet pestle (Sigma Aldrich, Denmark). The homogenate was sonicated for 45 s and centrifuged at 13,000 rpm for 10 min to collect supernatant. Protein quantification in the supernatant was carried out using bicinchoninic acid (BCA) Protein Assay kit (Pierce, Thermo Scientific, USA).

Ten µg protein were diluted with 4 × Laemmli sample buffer (Bio-Rad, USA) and 50 mM dithiothreitol (DTT) and denatured at 70 °C for 10 min. Gels (4–12% Bis-Tris, Bio-Rad, USA) were loaded and separation was performed by electrophoresis followed by transfer onto polyvinylidene fluoride (PVDF) membranes (Merck Millipore, Ireland). The membranes were blocked for 2 h with 5% non-fat dry milk (Blotting-Grade, Bio-Rad, USA) in TBS-T. Membranes were cut into two at approximately 75 kDa marker and incubated overnight at 4 °C in blocking buffer with primary eNOS (upper part of the membrane, control for endothelium removal; 1:500; Abcam) or TMEM16A (upper part of the membrane; 1:500; Abcam) or GAPDH (lower part of the membrane; 1:5000; Cell Signalling Technology, USA) antibody. The next day, the membranes were washed in TBS-T and incubated with horse-radish-peroxidase conjugated secondary goat-anti-rabbit antibody (1:2000, Cell Signalling Technology) for 2 h at room temperature. After washings in TBS-T, protein was detected using a chemiluminescent imaging system, ImageQuant LAS 4000 (GE Healthcare Life Sciences, USA, https://www.cytivalifesciences.com/), quantified using ImageJ (v1.48, NIH, https://imagej.nih.gov/) and normalized to GAPDH from the same sample. Data are expressed as the percentage of mean value of the 2- to 3-month-old group and shown as median and interquartile range.

### Ca^2+^-fluorimetry

Ratiometric measurements of intracellular Ca^2+^ were made simultaneously with the isometric force measurements similarly to previously described^50^. Arterial segments mounted in myograph were loaded with Fura 2-acetoxymethyl ester (2.5 µM; Fura 2/AM) in DMSO with 0.1% (wt/vol) cremophor and 0.02% (wt/vol) pluronic F127 for 2 h.

Arteries were excited by a 75 W xenon light source alternately at 340 and 380 nm, and emitted light was measured at 515 nm. Background fluorescence was determined before loading and subtracted from the measurements. Fluorescence was collected and stored digitally using Felix32 software (version 1.2, Photon Technology, USA, https://pti-felix32.software.informer.com/). Intracellular Ca^2+^ was expressed as the ratio of fluorescence during excitation at 340 nm and 380 nm.

### pH-fluorimetry

Ratiometric measurements of pH_i_ were made simultaneously with isometric force of the artery mounted in myograph as described previously^30^. Arterial preparation was loaded with 5 µmol/L AM-form of pH-sensitive fluorophore 2′,7′-bis-(2-carboxyethyl)-5-(and-6)-carboxyfluorescein (BCECF) in 0.02% DMSO for approximately 30 min at 37 °C. The excitation fluorescence ratio was recorded in BCECF-loaded arteries as the 510-nm emission during alternating 440- and 495-nm light excitation. The collected signal was converted to estimates of pH_i_ using a high-K^+^-nigericin technique^51^.

### Solutions

1. PSS for vessel isolation, in mM: 145 NaCl, 4.5 KCl, 1.2 NaH_2_PO_4_, 1 MgSO_4_, 0.1 CaCl_2_, 0.025 EDTA, 5 HEPES.
2. PSS for myograph experiments, in mM: 120 NaCl, 26 NaHCO_3_, 4.5 KCl, 1.2 NaH_2_PO_4_, 1.0 MgSO_4_, 1.6 CaCl_2_, 5.5 d-Glucose, 0.025 EDTA, 5 HEPES; equilibrated with 5% CO_2_ in 95% O_2_;
3. Cl^−^-free PSS, in mM: 120 sodium aspartate, 26 NaHCO_3_, 4.5 potassium aspartate, 1.2 NaH_2_PO_4_, 1.0 MgSO_4_, 1.6 CaSO_4_, 5.5 d-Glucose, 0.025 EDTA, 5 HEPES; equilibrated with 5% CO_2_ in 95% O_2_.
4. TBS-T: 50 mM Tri-HCl, 150 mM NaCl, pH 7.6 with 0.1% Tween.
5. Pierce lysis buffer: 25 mM Tris-HCl pH 7.4, 150 mM NaCl, 1 mM EDTA, 1% NP-40, 5% glycerol and Halt Protease Inhibitor cocktail (1:100; Thermo Scientific, USA).
6. High-K^+^-nigericin for pH_i_ measurements, in mM; 14 NaCl, 95 KCl, 0.4 NaH_2_PO_4_, 1.6 Na_2_HPO_4_, 2.6 Na-Gluconate, 1 MgSO_4_, 1.3 CaCl_2_, 20 NMDG-Cl, 25 HEPES, 5.5 d-Glucose, equilibrated with 100% O_2_.

### Drugs

All drugs were obtained from Sigma. Stock solutions of noradrenaline, acetylcholine and methoxamine (10 mM for all) were prepared using H_2_O and stock solution of MONNA was dissolved in DMSO. DIDS (1 mM) was dissolved in warm (37 °C) normal PSS immediately before use.

### Statistical analysis

The normality of data distribution was studied using the Shapiro–Wilk test (GraphPad Prism 7.0). Data are presented as mean ± SD (standard deviation, if data distribution is normal) or median ± the interquartile ranges (if data distribution is different from normal), *n* represents the number of animals (isometric force and membrane potential measurements) or number of samples (qPCR and Western blotting). Differences between concentration–response relationships to methoxamine were evaluated using repeated measures ANOVA. Statistical analyses of vessel diameter, force, relative area under CRC, pD_2_, membrane potential, mRNA and protein contents were performed using a two-sided unpaired Student’s t-test (for normally distributed data) or Mann–Whitney U-test (data distribution is different from normal). The differences were accepted as statistically significant if the *p*-value was < 0.05.

## Supplementary information

Supplementary Information.

## Data Availability

The data that support the findings of this study are available from the corresponding author upon reasonable request.
