# Supplementary material for: Trophic sympathetic influence weakens pro-contractile role of Cl− channels in rat arteries during postnatal maturation
Source: Sci Rep. 2020 Nov 17;10:20002. doi: 10.1038/s41598-020-77092-0 (PMC7673994; doi:10.1038/s41598-020-77092-0)
Supplement: Supplementary file 1 — Supplementary Information. [file 41598_2020_77092_MOESM1_ESM.pdf]

Trophic sympathetic influence weakens pro-contractile role of Cl^- channels in rat arteries during postnatal maturation

Short title: Cl^- channels in developing vasculature

Daria S. Kostyunina[1]; Lin Zhang[2]; Anastasia A. Shvetsova[1]; Ekaterina K. Selivanova[1]; Olga S. Tarasova[1,3]; Vladimir V. Matchkov[2]; Dina K. Gaynullina[1]

[1] Lomonosov Moscow State University, Moscow, Russia.
[2] Department of Biomedicine, Aarhus University, Aarhus, Denmark
[3] Institute for Biomedical Problems, Russian Academy of Sciences, Moscow, Russia.

Present address of Daria S. Kostyunina is School of Medicine, Conway Institute, University College Dublin, Dublin, Ireland.

Present address of Lin Zhang is Exercise Physiology, Beijing Sport University, Room 403, Teaching Experiment Center, No.48, Xinxi Road, Haidian District, Beijing, China, 100084

Corresponding author: Dina K. Gaynullina, Ph.D.
Department of Human and Animal Physiology
Lomonosov Moscow State University,
Leninskie Gory 1-12, Moscow, Russia, 119234
Phone number: +74959391416, e-mail: Dina.Gaynullina@gmail.com

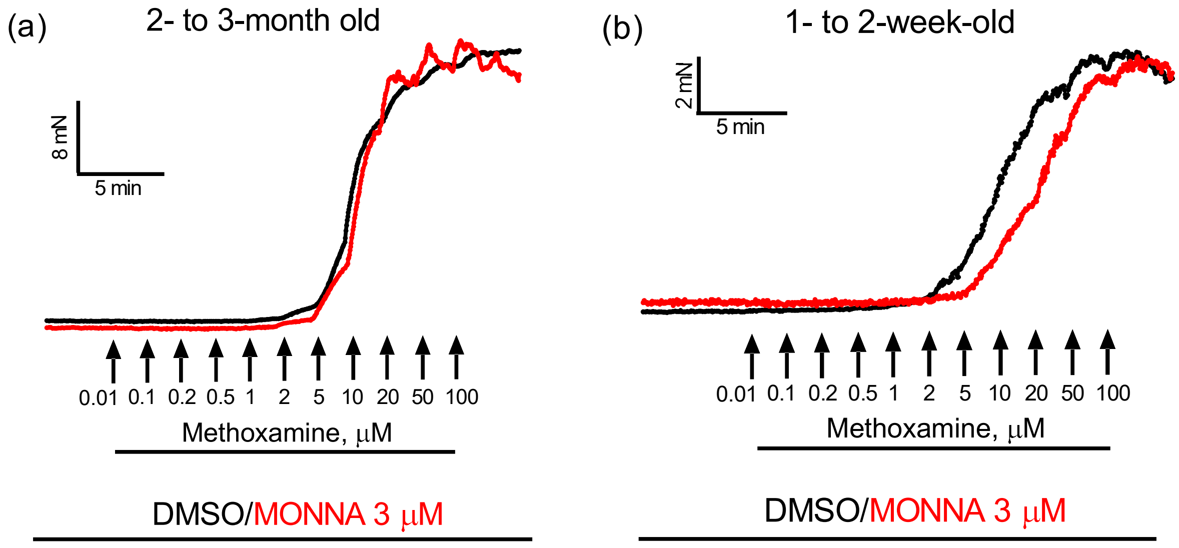

Figure S1. Representative traces for contraction of saphenous artery from 2- to 3-month-old (a) or 1- to 2-week-old (b) rats to methoxamine in the presence of DMSO or 3 μM MONNA as indicated.

Molecular weight markers
(membrane 2 for immunoblot with TMEM16A and eNOS antibodies; membrane 3 for immunoblot with GAPDH):

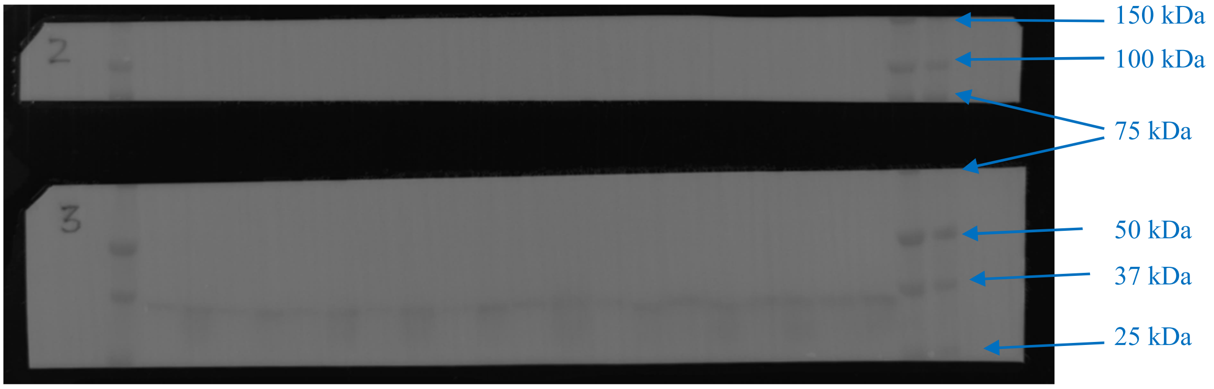

Western blot for TMEM16A:

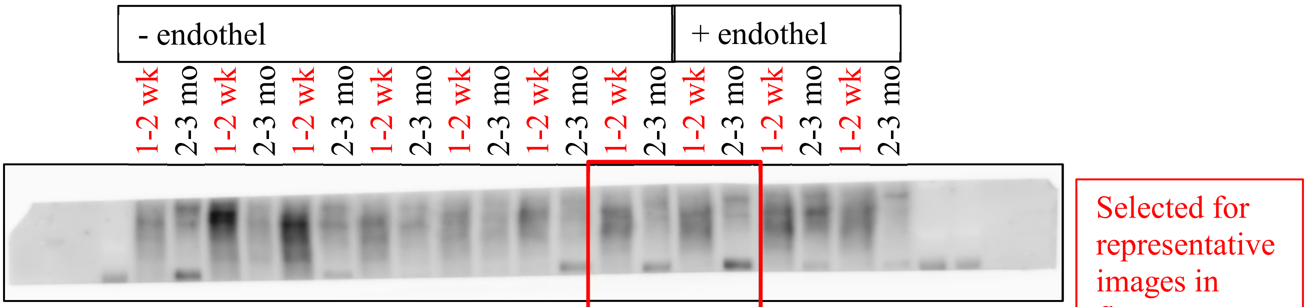

Western blot for eNOS:

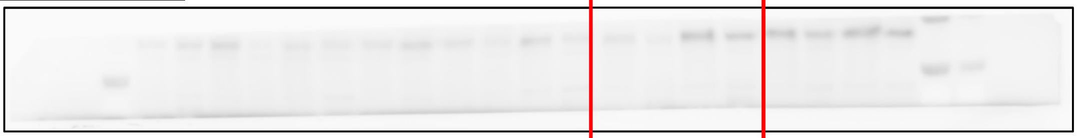

Western blot for GAPDH:

Western blot used for analyses, exposure time 20 sec:

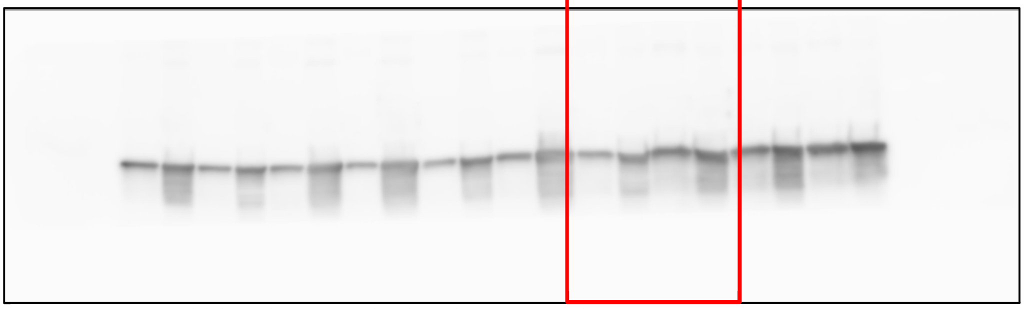

Over-exposed blot showing the border of membrane:

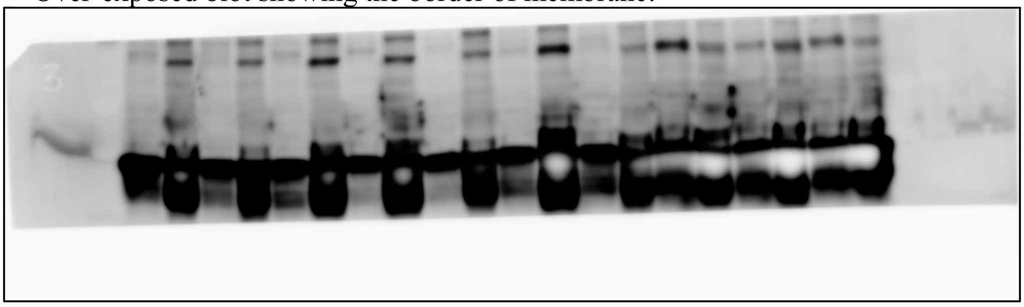

Figure S2. Full-length blots for detection of TMEM16A, eNOS and GAPDH. The membrane was cut at 150 kDa and at 75 kDa. The membrane above 150 kDa was discarded due to failed blotting for von Willebrand factor.

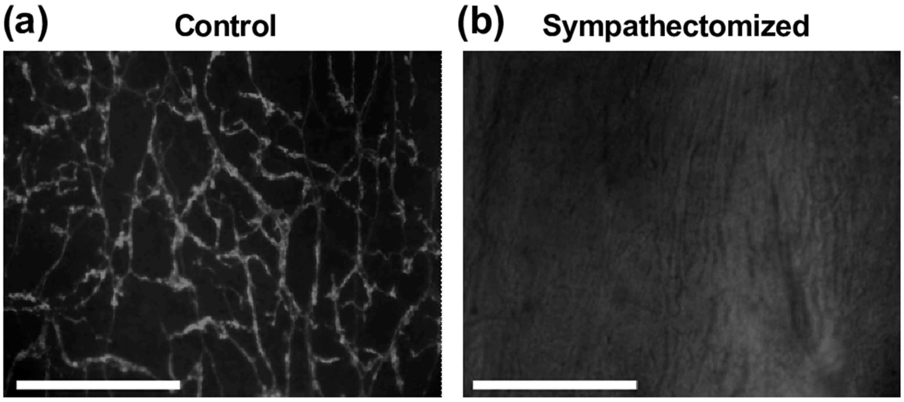

Figure S3. Chronic administration of guanethidine starting from the first postnatal day prevents the development of sympathetic innervation in the saphenous artery. Whole-mount preparations of saphenous arteries from control (vehicle-treated, a) and sympathectomized (b) rats, catecholaminergic nerve fibres are seen in light gray. Adrenergic nerve plexus was visualized using glyoxylic acid staining. The examples shown are representative of 3-4 independent experiments. Magnification x20, calibration bar – 100 µm.

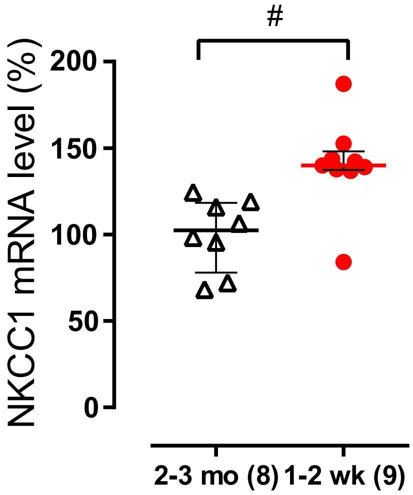

Figure S4. Expression of NKCC1 in endothelium-denuded arteries of two age-groups of rats. Data are expressed as a percentage of mean value of the 2- to 3-month-old group and shown as median and interquartile range. # $P < 0.05$ (Mann Whitney U test). Numbers in parentheses indicate the number of tissue samples.
